# Continuity of Care, Structural Inequities and Neonatal Outcomes among Pregnant Women with Sickle Cell Disease in Uganda: A Mixed-Methods Study

**DOI:** 10.21203/rs.3.rs-10153606/v1

**Published:** 2026-10-06

**Authors:** Jackline Akello, Savio Mwaka, Annettee Nakimuli, Grace Ndeezi, Musa Sekikubo, Ian Munabi, Ruth Namazzi, Deogratias Munube, David Mukunya, Pebalo Francis, Kenneth Mugabe, Sarah Kiguli, Joseph Rujumba

**Affiliations:** Makerere University; Infectious Diseases Research Collaboration; Makerere University; Makerere University; Makerere University; Makerere University; Makerere University; Mulago Hospital; Busitema University; Gulu University; Mbale Regional Referral Hospital; Makerere University; Makerere University

**Keywords:** Sickle cell disease, continuity of care, reproductive justice, maternal health, neonatal outcomes

## Abstract

Pregnancy among women with sickle cell disease (SCD) is high-risk and shaped by clinical vulnerability and systemic barriers. We examined how continuity of care, equity, and social support influence neonatal outcomes and reproductive experiences among women with SCD in Uganda. We conducted a convergent mixed-methods study among 140 enrolled pregnant women with SCD, of whom 135 completed follow-up and were included in the final analysis. Quantitative data included measures of continuity of care (antenatal attendance, follow-up adherence, ease of access), equity indicators (education, employment, marital status, partner support), and clinical vulnerability (haemoglobin, Vaso-occlusive crises). Logistic regression and generalized structural equation modelling assessed associations and mediation. Qualitative data from 34 in-depth interviews and 5 focus group discussions with women and healthcare providers were analysed thematically and integrated with quantitative findings. Continuity of care was strongly protective against adverse fetal or neonatal outcomes (AOR 0.48, 95% CI 0.27–0.87). Adequate antenatal attendance, ease of access, and consistent follow-up adherence were individually protective. Clinical severity, particularly at least three Vaso-occlusive crises, substantially increased risk (AOR 6.33, 95% CI 1.18–33.96). Education, employment, and marital status were not significantly associated with outcomes. Partner support was associated with higher continuity scores (β 0.65, 95% CI 0.13–1.16). Qualitative findings converged with quantitative results, revealing threats to reproductive autonomy, stigma and social abandonment, structural health system barriers (costs, referrals, shortages), and provider moral distress. These systemic constraints undermined care continuity and compounded clinical risk. Continuity of care is a critical protective factor for neonatal outcomes among women with SCD, while socio-demographic advantage alone does not confer protection. Policies should prioritize strengthening care continuity, enhancing social support, and addressing structural health system inequities to improve outcomes in this high-risk population.

## Introduction

Sickle cell disease (SCD) is a chronic inherited haemoglobinopathy that disproportionately affects populations of African descent and represents a significant contributor to maternal and perinatal morbidity and mortality in sub-Saharan Africa(Nessar et al. 2025). Globally, over 300,000 infants are born annually with SCD, the majority in Africa (Piel et al. 2013). In Uganda, approximately 13% of the population carries the sickle cell trait, placing thousands of women of reproductive age at risk of pregnancy-related complications associated with SCD (Ndeezi et al. 2016). Pregnant women with SCD face increased risks of hypertensive disorders, severe anaemia, Vaso-occlusive crises, preterm birth, stillbirth, and maternal death(Cells et al. 2015) (Corsia et al. 2025). While these biomedical risks are well documented, far less attention has been paid to the social, structural, and rights-based dimensions shaping pregnancy experiences among women with SCD in low-resource settings.

Evidence from sub-Saharan Africa demonstrates that maternal outcomes are strongly influenced by health system factors, including delayed antenatal care, referral fragmentation, financial barriers, and inequitable quality of care (O. M. R. Campbell and Graham 2006)(Kruk et al. 2018). For women with chronic conditions such as SCD whose pregnancies require coordinated, multidisciplinary management, these systemic weaknesses may amplify underlying clinical vulnerability.

Reproductive health is internationally recognized as a fundamental human right. The International Conference on Population and Development (ICPD) Programme of Action affirms the right of all individuals to decide freely and responsibly the number and spacing of their children and to access information and services free from discrimination, coercion, or violence(Dennis-Antwi BSN RNM PHN FWACN STTI et al. 2008).However, women with chronic or genetic conditions frequently encounter implicit or explicit constraints on reproductive autonomy, including discouragement from childbearing, moral judgment, and differential treatment in healthcare settings (Luna and Luker 2013).

The Reproductive Justice framework extends beyond formal legal rights to emphasize the social, economic, and political conditions necessary for meaningful reproductive autonomy, particularly among marginalized populations. Within African contexts, scholars have argued for adaptations of reproductive justice that explicitly address colonial legacies, structural underinvestment in health systems, and gendered power relations shaping reproductive decision-making (Thomson et al. 2023).

In Uganda, women with SCD navigate pregnancy within a context of constrained blood supply systems, out-of-pocket expenditures, referral delays, stigma, and gender inequities. Emerging qualitative evidence suggests that stigma from partners, families, communities, and even healthcare providers contribute to delayed care-seeking, treatment interruptions, and psychological distress. Yet few studies have examined how these lived experiences intersect with measurable clinical outcomes, leaving important gaps in understanding how structural inequities translate into adverse maternal and neonatal outcomes.

This study adopts a social justice and reproductive rights lens to examine pregnancy among women with SCD in Uganda. By integrating prospective cohort data with in-depth qualitative findings, we move beyond a purely biomedical narrative to illuminate how continuity of care, social equity, reproductive autonomy, and health system structures interact to shape both experiences and outcomes. This approach aligns with the World Health Organization’s call for equity-cantered, quality maternal health systems that ensure respectful, rights-based care for vulnerable populations (World Health Organization 2016).

## Methods

### Study Design

We conducted a convergent parallel mixed-methods study, in which quantitative and qualitative data were collected concurrently, analysed separately, and then integrated during interpretation. We employed a phenomenological qualitative design using in-depth interviews (IDIs) and focus group discussions (FGDs) to explore lived experiences, social inequities, reproductive autonomy, and health system factors shaping pregnancy among women with sickle cell disease (SCD). This approach was grounded in reproductive justice and rights-based frameworks, enabling examination of both individual experiences and structural determinants of reproductive health. This approach enabled a comprehensive examination of both measurable outcomes and lived experiences related to pregnancy, social equity, and reproductive rights among women with SCD.

Quantitatively, we conducted a prospective cohort study among 140 pregnant women with confirmed SCD receiving care at the two tertiary hospitals. Participants were enrolled during pregnancy and followed through delivery to assess fetal and neonatal outcomes.

### Participants

Eligible participants were pregnant women aged 18 years or older with laboratory-confirmed SCD (HbSS) attending antenatal care or referred for obstetric management. Women were consecutively recruited at presentation to minimize selection bias. Written informed consent was obtained prior to participation.

### Study Setting

The study was conducted at two tertiary hospitals in Uganda: Kawempe National Referral Hospital (KNRH) in Kampala and Mbale Regional Referral Hospital (MRRH) in eastern Uganda. These hospitals serve large catchment populations and are major centres for maternal care and sickle cell services, providing an appropriate context for examining systemic and equity-related issues.

### Quantitative Component

#### Study Population

The quantitative cohort consisted of 140 pregnant women aged 16 years and above with confirmed sickle cell disease (HbSS,) who received antenatal care and or delivered at either study site between October 2024 and October 2025.

#### Eligibility Criteria

Inclusion criteria involved pregnant women with confirmed diagnosis of SCD by haemoglobin (Hb) electrophoresis, aged ≥ 16 years, received antenatal care and or delivered at one of the study hospitals and willing to provided informed consent. Women referred in after delivery or those unwilling or unable to provide informed consent were excluded although none declined to give consent during the study.

### Data Collection

Baseline socio-demographic and clinical data were collected using structured questionnaires and medical record review. Equity indicators included education level, employment status, marital status, and partner involvement in care. Clinical vulnerability measures included haemoglobin level during pregnancy, number of Vaso-occlusive crises, and hypertensive disorders.

Continuity of care was assessed using a composite score (range 0–4), derived from four components: (1) adequate antenatal care attendance (≥ 4 visits), (2) perceived ease of access to services, (3) follow-up frequency (at least monthly), and (4) follow-up adherence (always/often compliant). Each component contributed one point to the overall score.

### Outcome Measure

The primary outcome was a composite adverse fetal or neonatal outcome, including abortions, stillbirth, admission to NICU and early neonatal deaths as documented in delivery records.

### Statistical Analysis

Descriptive statistics summarized participant characteristics. Logistic regression models estimated associations between continuity of care, equity indicators, clinical vulnerability, and adverse outcomes. Multivariable models adjusted for socio-demographic and clinical covariates. Linear regression assessed predictors of continuity of care. Mediation analysis was conducted using generalized structural equation modelling (GSEM) with robust standard errors. Statistical significance was defined as p < 0.05.

### Qualitative Component

#### Study Population

The qualitative sample included pregnant and postpartum women with SCD who had received care at either study site and Healthcare providers involved in the care of pregnant women with SCD, including obstetricians, midwives, medical officers, anaesthesia providers, and nurses

#### Sampling Strategy

Purposive sampling with maximal variation was used to ensure diversity by age, parity, marital status, education level, socioeconomic status, pregnancy outcome, and healthcare provider cadre. Women were recruited from antenatal wards, postnatal wards, sickle cell clinics, and inpatient units. Clinical staff introduced eligible participants to the research team, after which trained qualitative researchers provided study information and obtained informed consent. Recruitment continued iteratively until thematic saturation was achieved, where no new concepts, categories, or relationships emerged from successive interviews.

Healthcare providers were sampled purposively to ensure representation across professional cadres and roles, including Obstetricians and gynaecologists, Midwives and nurses, medical officers, and Anaesthesia providers. Participants were recruited through departmental meetings and direct invitations. Sampling aimed to capture variation in years of experience, facility role, and exposure to women with SCD in pregnancy. Recruitment continued until data saturation was reached across cadres and facility sites.

#### Data Collection and management

We conducted 15 IDIs and 2 FGDs with women with SCD and 19 IDIs and 3 FGDs with healthcare providers and saturation was achieved. Semi-structured interview guides explored reproductive intentions and decision-making, experiences of pregnancy and care-seeking, perceived stigma, discrimination, and moral judgment, interactions with healthcare providers, support systems and health system constraints. Interviews were conducted in private settings, audio-recorded, transcribed verbatim, and translated into English where necessary. The data was De-identified, assigned unique study codes and stored securely on password-protected servers. Field notes were taken during and immediately after interviews to capture contextual details, non-verbal cues, and reflexive observations. Weekly analytic meetings were held where transcripts were reviewed and emerging codes discussed. To minimize social desirability bias and power imbalances, the principal investigator who is known to both sites did not take part in the interviews.

#### Qualitative Analysis

Data were analysed using both inductive and deductive thematic analysis following Braun and Clarke’s six-step framework(Braun and Clarke 2006): familiarization with data, initial code generation, reaching for themes, reviewing themes, refining and naming themes, producing the report. Coding was guided by reproductive justice and reproductive rights frameworks. A codebook was developed iteratively and refined through team discussions. NVivo software was used to manage data. To enhance rigor, we employed investigator triangulation, reflexive journaling and member checking.

#### Integration of Quantitative and Qualitative Data

Integration occurred at the interpretation stage using triangulation and joint displays. Quantitative and qualitative findings were compared to identify convergence, complementarity, and divergence. This approach allowed us to link statistical associations with lived experiences and structural contexts.

## Results

### Quantitative Findings

Figure 1 presents participant flow and follow-up status in the cohort study of pregnant women with sickle cell disease. A total of 140 pregnant women with sickle cell disease were enrolled at baseline and all were followed during the study period. Of these, 5 participants were lost to follow-up, while 135 participants (96.4%) completed follow-up and were included in the final analysis. This high retention rate minimized the potential for attrition bias in the estimation of study outcomes.

### Participant Characteristics

Table 1 presents the baseline characteristics of the 135 pregnant women with sickle cell disease that were included in the study. The mean age was 24.2 years (SD 4.8), with mean gravidity 1.78 (SD 0.93) and parity 0.54 (SD 0.85). The mean haemoglobin level at enrolment was 7.75 g/dL (SD 1.86). Participants attended an average of 3.79 antenatal care (ANC) visits (SD 1.78), and the mean continuity of care score was 2.46 (SD 1.15) on a 0–4 scale.

Just over half had attained secondary education or higher (54.1%), 34.1% were employed, and 51.1% were married. Approximately half (51.1%) were enrolled through routine ANC services, while 48.9% were referrals in. Partner involvement in care was reported by 44.4% of participants.

Clinically, 78.5% of women experienced at least one Vaso-occlusive crisis during pregnancy, and 12.6% developed hypertension or preeclampsia. Overall, 31.9% experienced a non-live birth outcome. Among live births, 69.6% of neonates required NICU admission, and 79.3% of pregnancies were classified as having an adverse fetal or neonatal outcome.

### Continuity of Care

Table 2 presents the parameters for continuity of care scores where approximately half of participants (50.7%) attended at least four ANC visits, and 51.1% reported easy access to services. Nearly all (97.0%) reported at least monthly follow-up, although consistent follow-up compliance (always/often) was reported by 47.4%.

### Factors associated with adverse fetal and neonatal outcomes

Table 3 presents crude and adjusted associations between equity indicators, continuity measures, clinical vulnerability factors, and adverse outcomes. In bivariate analyses, higher continuity of care, attending ≥ 4 ANC visits, ease of access, and follow-up compliance were associated with lower odds of adverse outcomes, while frequent Vaso-occlusive crises were associated with increased risk. In multivariable analysis, continuity of care remained independently associated with reduced odds of adverse outcomes (AOR 0.48, 95% CI 0.27–0.87). Women experiencing ≥ 3 Vaso-occlusive crises had significantly higher odds of adverse outcomes (AOR 6.33, 95% CI 1.18–33.96). Equity indicators were not significantly associated with outcomes after adjustment.

### Continuity as a mediator

In the mediator model, partner involvement in care was the only equity indicator significantly associated with higher continuity scores (β 0.65, 95% CI 0.13–1.16), while other equity and clinical factors were not associated. The mediator model explained a modest proportion of variability in continuity (R^2^ = 0.19). Higher continuity of care was associated with lower odds of adverse fetal or neonatal outcomes (OR 0.48, 95% CI 0.27–0.87). Mediation analysis showed no statistically significant indirect effects of education, employment, or marital status through continuity. Partner involvement demonstrated a suggestive indirect protective pathway via improved continuity (indirect OR 0.63, p = 0.077), although this did not reach statistical significance. Overall, these findings indicate that continuity of care plays a protective role, with limited evidence that equity indicators influence outcomes through this pathway. Table 4 presents Continuity of care score modelled as a continuous mediator using linear regression.

### Qualitative Findings

#### Theme 1: Reproductive Autonomy Under Threat

Many women reported being discouraged from pregnancy by healthcare providers, family members, or partners. Some were told they that they should not get pregnant while others were blamed for conceiving, leading to internalized stigma and delayed care-seeking. *They told me that my life can’t support a baby, so when I give birth to this baby, either I get long term family planning, or they can tie the tubes*. (SCD IDI1) Another respondent reported that one of the doctors said, *‘if you were my daughter, I wouldn’t allow you to get pregnant.’* (SCD IDI3). This undermines the reproductive rights of patients with sickle cell disease.

#### Theme 2: Stigma, Moral Judgment, and Social Abandonment

Women described stigma at multiple levels from communities, partners, and healthcare providers. Several reported abandonments by partners during pregnancy or hospitalization. Some of the respondents noted that within communities’ people showed them all sorts of gestures and pitied them and thought they were cursed. People often wondered why they conceived and openly criticized them. *It has been my grandmother taking care of me because my mother left me to my grandparents after giving birth to me and they raised me. (FGD-RECO3). Some are chased from home once they realize that their sickle cell daughter is pregnant. (FGD -RECO 5)*. One of the doctors stated that most times the patient had no family support and this made care difficult as certain decisions required close relatives. *She lacked family support. No one brought her food or money. She deteriorated quickly (HWK06)*.

#### Theme 3: Structural Barriers and Health System Inequities

Participants described financial constraints, long distances, blood shortages, and referral delays as major barriers. Healthcare providers acknowledged systemic failures, including lack of national guidelines and resource constraints. Some of the respondents observed that they were referred multiple times from one facility to another which was so frustrating and costly. Others explained that they had to forego meals or skip some of the antenatal care visits in order to meet the costs of referrals and also purchase some of the medical supplies. *I have sickle cell disease but the problem is that I come from far to come get treatment from here so the transport I use is much and not easy to get. (FGD -RECO10)*.

The least expensive drug costs four thousand shillings and yet it’s required as a full dose, so to get the full dose would cost about twenty thousand or twenty-five thousand. (FGD -RECO7)

I was referred to Women’s Hospital, and they charged us but covered half after I explained my financial situation. (SCD IDI1)

#### Theme 4: Moral Injury Among Providers

Healthcare providers reported emotional distress and moral injury due to their inability to provide equitable care, particularly when witnessing preventable suffering. Many Health providers reported feeling helpless and frustrated at times especially when there are shortages of blood, medical supplies and even at times cross consultation with other specialities like haematologists. The lack of clear management protocol or guidelines even frustrated the health workers the more. One of the health workers stated, *no folic acid, no morphine… how do we manage these crises without tools? (HW4 DM)*. Another Doctor from Kawempe shared, *you feel emotionally drained, especially if after doing your best, you lose a patient (HW17 DK)*. Medications weren’t in the pharmacy. She couldn’t afford to buy them herself (HW5 K FGD). These systemic shortages created a sense of helplessness between both the health care providers and the patients.

### Convergent Integration of Quantitative and Qualitative Findings

The quantitative and qualitative findings converge to highlight continuity of care as a key determinant of neonatal outcomes. Continuity measures were strongly protective against adverse outcomes, and qualitative data elucidate the barriers that undermine care engagement, including structural, financial, and social challenges. Partner support emerged as a facilitator of continuity, aligning with qualitative reports of social support mitigating isolation and improving care adherence. Clinical vulnerability remains a strong driver of risk, consistent with provider narratives of moral distress when unable to manage severe complications. Importantly, socio-demographic advantages alone did not confer protection, reinforcing qualitative evidence of provider-imposed reproductive restrictions and community stigma.

Together, these results underscore the interplay of structural inequities, continuity of care, and social support in shaping both reproductive experiences and outcomes among women with SCD in Uganda. Interventions to strengthen continuity, enhance partner/family involvement, and address system-level barriers are critical for improving maternal and neonatal outcomes in this high-risk population.

## Discussion

This mixed-methods study demonstrates that continuity of care is a critical and independent protective factor against adverse fetal and neonatal outcomes among women with SCD in Uganda. While biomedical vulnerability particularly recurrent Vaso-occlusive crises substantially increased risk, sustained engagement in care significantly mitigated adverse outcomes even after adjustment for socio-demographic and clinical factors. These findings are consistent with global evidence that quality and continuity of maternal care are central determinants of perinatal survival, particularly among high-risk pregnancies (Cells et al. 2015).

Continuity of care is increasingly recognized as a core dimension of health system quality (Smith-Whitley 2019). The World Health Organization Quality of Care Framework emphasizes effective referral, coordinated management, and respectful care as essential for improving maternal and neonatal outcomes (Kruk et al. 2018). Our findings extend this framework by demonstrating that, in a clinically vulnerable population such as women with SCD, continuity is not merely a quality indicator but a protective clinical mechanism(Grismore, Roberts, and Montgomery 2025).

In contrast, conventional equity indicators such as education and employment were not independently associated with neonatal outcomes. This finding aligns with reproductive justice scholars, who argue that health outcomes are shaped not only by individual socio-economic status but by structural conditions that enable or constrain access to care (Kruk et al. 2018)(O. M. Campbell and Graham 2006). As articulated by Sister Song Women of Colour Reproductive Justice Collective, reproductive rights must be understood within the broader context of social, political, and economic inequities (Kruk et al. 2018). In settings where health systems are fragmented or under-resourced, individual-level advantages may not translate into improved outcomes.

Partner involvement was associated with higher continuity of care scores, consistent with evidence from sub-Saharan Africa showing that male partner engagement improves antenatal attendance and adherence(Angusubalakshmi, Boratne, and Venkataraman 2023). However, mediation analysis suggested that broader social equity factors alone did not directly protect against adverse outcomes, reinforcing the importance of structural health system capacity.

The qualitative findings further contextualize these results. Women described stigma, discouragement from pregnancy, and moral judgment from providers and communities. Similar patterns have been documented among women with chronic or genetic conditions globally, where reproductive autonomy is often undermined by paternalistic counselling (Thomson et al. 2023; Brown et al. 2010). In Uganda, where SCD prevalence remains high, such stigma may intersect with systemic maternal health challenges, including blood shortages and referral delays.

Healthcare providers reported emotional distress and helplessness in the face of systemic shortages. This aligns with emerging literature on moral distress and “moral injury” among healthcare workers operating in resource-constrained environments (Forbes Shepherd et al. 2022; Ross and Solinger 2025). When clinicians lack essential tools such as blood products, analgesics, or clinical guidelines, they experience ethical strain that can compromise workforce morale and quality of care.

Together, these findings suggest that improving outcomes for pregnant women with SCD requires policy interventions that strengthen continuity across the care pathway: early risk stratification, integrated obstetric–haematology management, reliable blood supply systems, and reduced financial barriers. Uganda’s dual burden of communicable and non-communicable diseases underscores the need to integrate SCD management within national maternal health strategies (Ross and Solinger 2025).

By linking quantitative outcome modelling with qualitative insights into lived experience and provider constraints, this study demonstrates that reproductive autonomy, clinical vulnerability, and system capacity are deeply interconnected. A rights-based approach anchored in continuity of care and structural equity is essential to advancing maternal and neonatal survival among women with SCD.

### Implications for Policy and Practice

Continuity of care is a key protective factor for neonatal outcomes among women with SCD and should be prioritized within high-risk maternal health services. National policy should institutionalize structured care pathways for pregnant women with SCD, including early risk identification, coordinated obstetric–haematology co-management, and clear referral protocols supported by national clinical guidelines.

Reducing financial and geographic barriers is essential to sustaining care engagement. Transport costs, medication shortages, and fragmented referrals undermine continuity. Strengthening financial protection, ensuring availability of essential medicines and blood products, and improving referral coordination are critical system-level interventions.

Partner involvement was associated with improved continuity, suggesting that maternal health programmes should incorporate family engagement and couple-focused counselling to enhance sustained utilization of services.

Provider-reported moral distress reflects systemic weaknesses, including supply shortages and unclear protocols. Addressing these constraints through strengthened supply chains, workforce support, and standardized management pathways will improve both provider capacity and patient outcomes. A rights-based health systems approach cantered on continuity, structural equity, and supportive care environments is necessary to improve maternal and neonatal outcomes among women with SCD.

### Strengths and Limitations

The strengths of our study lie in the mixed-methods design, prospective cohort data, inclusion of both patient and provider perspectives, and grounding in rights-based frameworks while the limitations include potential social desirability bias, single-country setting, and limited generalizability beyond similar contexts.

## Conclusion

Continuity of care is a critical protective factor for neonatal outcomes among women with SCD, while socio-demographic advantage alone does not confer protection. Policies should prioritize strengthening care continuity, enhancing social support, and addressing structural health system inequities to improve outcomes in this high-risk population.

## Figures and Tables

**Figure 1:**
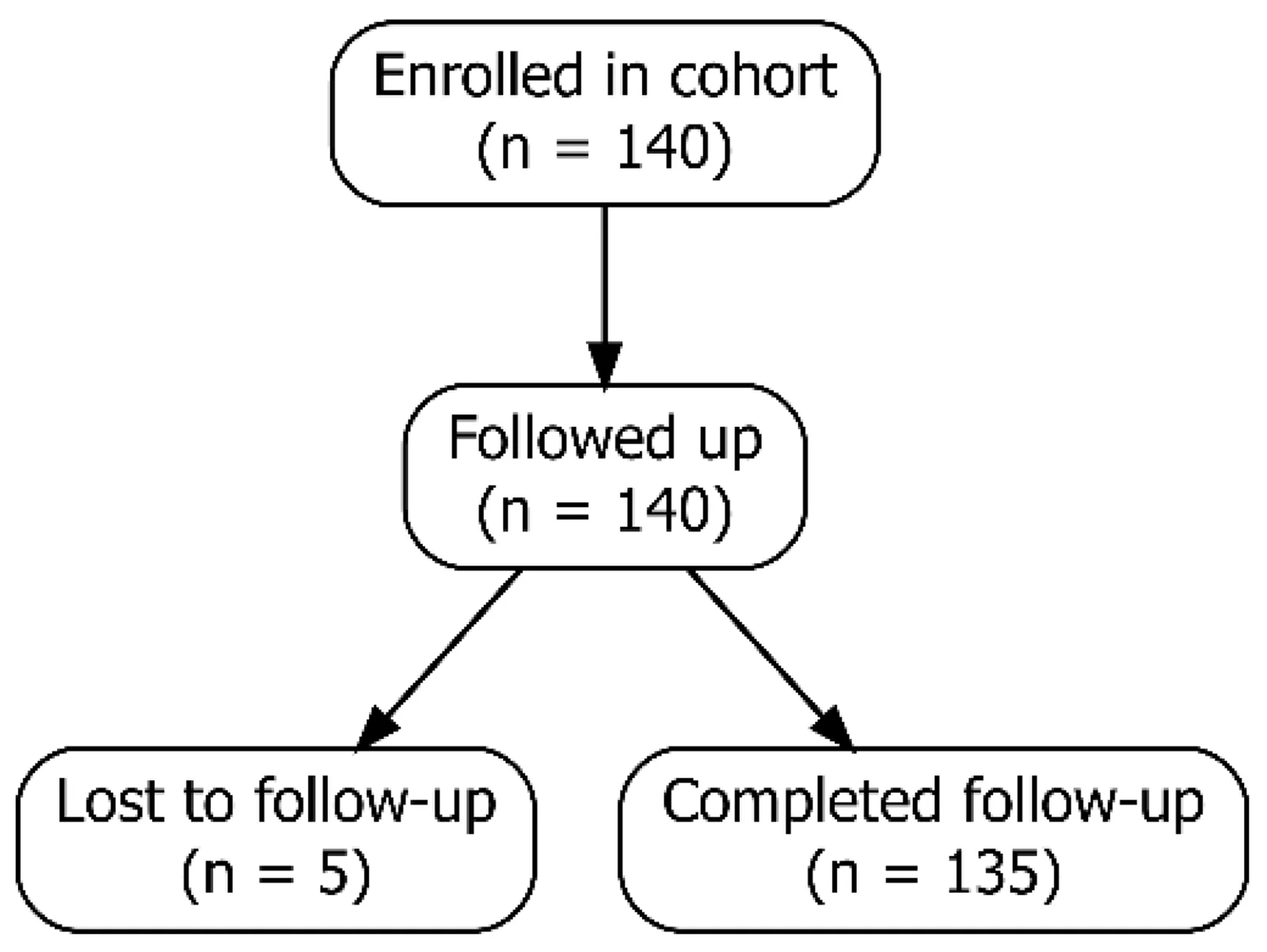
Participant flow and follow-up status in the cohort study of pregnant women with SCD

**Table 1:** Baseline Characteristics of the participants for the study

Characteristic, n = 135	N	Summary, mean (SD); median (IQR)
Continuous variables		
Age (years)	135	24.22 (4.75); 23.0 [21.0–27.0]
Gravidity	135	1.78 (0.93); 2.0 [1.0–2.0]
Parity	126	0.54 (0.85); 0.0 [0.0–1.0]
Hemoglobin (g/dL)	113	7.75 (1.86); 7.80 [6.50–8.80]
Total ANC visits attended	134	3.79 (1.78); 4.0 [3.0–5.0]
Continuity of care score (0–4)	135	2.46 (1.15); 3.0 [1.0–3.0]
Categorical variables		n(%)
**Education**		
Primary or less	135	62 (45.9)
Secondary+	135	73 (54.1)
**Employment status**		
Not employed	135	89 (65.9)
Employed	135	46 (34.1)
**Marital status**		
Not married	135	66 (48.9)
Married	135	69 (51.1)
**Enrollment type**		
Known from site ANC	135	69 (51.1)
Referred in	135	66 (48.9)
**Partner support included**		
No partner support	135	75 (55.6)
Partner included	135	60 (44.4)
**Vaso-occlusive crises during pregnancy**		
None	135	29 (21.5)
1–2 episodes	135	71 (52.6)
≥3 episodes	135	35 (25.9)
**Development of hypertension/preeclampsia**		
No	135	118 (87.4)
Yes	135	17 (12.6)
**Non-live birth**		
No	135	92 (68.2)
Yes	135	43 (31.9)
**NICU admission (live births only)**	92	
No	92	28 (30.4)
Yes	92	64 (69.6)
**Adverse fetal/neonatal outcome**	135	
No	135	28 (20.7)
Yes	135	107 (79.3)

**Table 2:** Continuity of Care Parameters and scores

Measure	Category	n (%)
ANC visits ≥ 4	No	66 (49.3)
	Yes	68 (50.7)
Access to services easy	No	66 (48.9)
	Yes	69 (51.1)
Follow-up frequency ≥ monthly	No	4 (3.0)
	Yes	131 (97.0)
Follow-up compliance (always/often)	No	71 (52.6)
	Yes	64 (47.4)
Continuity of care score (0–4)	0	2 (1.5)
	1	35 (25.9)
	2	28 (20.7)
	3	39 (28.9)
	4	31 (23.0)
Continuity score summary	Mean (SD)	2.46 (1.15)
	Median [IQR]	3 [1–3]

**Table 3:** Crude and adjusted associations between equity indicators, continuity measures, clinical vulnerability factors, and adverse outcomes.

Variable	COR (95% CI)	p-value	AOR (95% CI)	p-value
**Equity indicators**				
Secondary education+	0.86 (0.37–1.98)	0.714	1.53 (0.58–4.01)	0.39
Employed	0.91 (0.38–2.18)	0.837	0.99 (0.33–2.96)	0.989
Married	0.88 (0.38–2.03)	0.77	1.09 (0.35–3.35)	0.884
Partner included	0.75 (0.33–1.74)	0.507	1.24 (0.31–5.02)	0.76
Referred in labor	0.95 (0.41–2.17)	0.895	0.86 (0.34–2.17)	0.744
**Continuity indicators**				
Continuity score (per unit)	0.47 (0.30–0.73)	0.001	0.48 (0.27–0.87)	0.016
ANC ≥ 4 visits	0.27 (0.10–0.68)	0.006		
Access to services easy	0.27 (0.11–0.69)	0.006		
Follow-up ≥ monthly	1.28 (0.13–12.84)	0.832		
Compliance always/often	0.34 (0.14–0.83)	0.018		
**Clinical vulnerability**				
Hemoglobin (per g/dL)	0.83 (0.64–1.07)	0.145	0.92 (0.70–1.21)	0.572
VOC 1–2 episodes	3.15 (1.21–8.17)	0.018	2.53 (0.85–7.52)	0.094
VOC ≥ 3 episodes	7.53 (1.87–30.39)	0.005	6.33 (1.18–33.96)	0.031
Hypertension/preeclampsia	2.12 (0.46–9.87)	0.339	1.10 (0.19–6.50)	0.913

**Table 4:** Continuity of care score modelled as a continuous mediator using linear regression

Predictor / Pathway	Effect type	Estimate (95% CI)	p-value
Predictors of continuity (mediator model)			
Secondary education+	Effect on continuity	0.34 (− 0.13 to 0.80)	0.152
Employed	Effect on continuity	0.38 (− 0.11 to 0.87)	0.131
Married	Effect on continuity	−0.05 (− 0.58 to 0.48)	0.861
Partner included	Effect on continuity	0.65 (0.13 to 1.16)	0.014
Referred in labor	Effect on continuity	−0.02 (− 0.48 to 0.43)	0.917
Hemoglobin (per g/dL)	Effect on continuity	0.04 (− 0.07 to 0.16)	0.457
VOC 1–2 episodes	Effect on continuity	−0.27 (− 0.77 to 0.23)	0.281
VOC ≥ 3 episodes	Effect on continuity	−0.49 (− 1.10 to 0.11)	0.109
Hypertension/preeclampsia	Effect on continuity	0.02 (− 0.58 to 0.62)	0.949
Mediator → outcome			
Continuity score (per 1-point increase)	Total association (OR)	0.48 (0.27–0.87)	0.016
Education (Secondary + vs Primary or less)			
	Indirect (via continuity)	0.78 (0.55–1.12)	0.179
	Direct	1.53 (0.58–4.01)	0.39
	Total	1.20 (0.43–3.30)	0.73
Employment (Employed vs Not employed)			
	Indirect (via continuity)	0.76 (0.50–1.14)	0.186
	Direct	0.99 (0.33–2.96)	0.989
	Total	0.75 (0.25–2.30)	0.619
Marital status (Married vs Not married)			
	Indirect (via continuity)	1.04 (0.72–1.49)	0.856
	Direct	1.09 (0.35–3.35)	0.884
	Total	1.13 (0.36–3.50)	0.839
Partner support (Partner included vs none)			
	Indirect (via continuity)	0.63 (0.37–1.05)	0.077
	Direct	1.24 (0.31–5.02)	0.76
	Total	0.78 (0.22–2.76)	0.696

Continuity of care score modeled as a continuous mediator using linear regression with robust standard errors. Adverse fetal/neonatal outcome modeled using logistic regression within a generalized structural equation model. Models adjusted for enrollment pathway, hemoglobin level, vaso-occlusive crises, and hypertensive disorders.

## Data Availability

The datasets generated and/or analysed during the current study are publicly available in a public repository via the link https://doi.org/10.7910/DVN/F3BVEZ.

## Key message

- Continuity of care is a strong protective factor against adverse fetal and neonatal outcomes among women with SCD in Uganda.
- Socio-demographic advantages alone do not protect against poor outcomes.
- Partner support enhances engagement with care, contributing to higher continuity of care and potentially mitigating adverse outcomes.
- Structural health system barriers and clinical disease severity interact to shape reproductive experiences and outcomes.
- Provider moral distress reflects systemic inadequacies, including shortages, referral delays, and lack of clear management protocols.
- A rights-based, health systems approach is needed, prioritizing strengthened continuity of care, social support mechanisms, and structural reforms to improve maternal and neonatal outcomes.
